# Are quantity and content of psychiatric interventions associated with suicide? A case-control study of a Swedish sample

**DOI:** 10.1186/s12888-019-2421-z

**Published:** 2020-01-09

**Authors:** Fredrik Holländare, Maria Tillfors, Axel Nordenskjöld, Tabita Sellin

**Affiliations:** 1 Department of Psychiatry, School of Medical Sciences, Örebro University, Örebro, Sweden; 2 Department of Social and Psychological Studies, Karlstad University, Karlstad, Sweden; 3 Centre for Health and Medical Psychology, Örebro University, Örebro, Sweden; 4 University Health Care Research Centre, Faculty of Medicine and Health, Örebro University, Örebro, Sweden

**Keywords:** Suicide, Suicide prevention, Psychiatry, Psychiatric interventions, Case-control

## Abstract

**Background:**

Research is required to identify those psychiatric interventions with a protective effect against suicide. The overarching aim of the current study was to examine whether completed suicide in psychiatric patients in a Swedish population was associated with the quantity and nature of previous medical and psychosocial treatment interventions.

**Methods:**

This retrospective case-control study (*n* = 308) compared a group of deceased psychiatric patients with matched controls. For every case of suicide, a control was found within psychiatry that matched according to sex, age, and primary psychiatric diagnosis. A stepwise forward logistic regression model with suicide as the dependent outcome variable was used.

**Results:**

Receiving pharmacotherapy combined with psychotherapy [OR: 0.44 (95% CI: 0.226–0.876), *p* = 0.019] and a higher number of outpatient visits in psychiatry [OR: 0.99 (95% CI: 0.982–0.999), *p* = 0.028] were negatively associated with suicide. These associations were still significant after controlling for previous serious suicide attempts and somatic comorbidity.

**Conclusions:**

Frequent visits and pharmacotherapy combined with psychotherapy seem to be important for preventing suicide in psychiatric patients. The reasons for not receiving such therapy are important issues for further study.

## Background

Over the past 20–30 years, suicide rates have declined overall in European countries with previously high rates of suicide, such as Denmark, Estonia, Germany, Hungary, and Sweden [1]. Ever since the adoption of a national suicide prevention strategy in 2008, [2] the suicide rate among males in Sweden has declined further [3]. The prevention strategy involves nine strategic action areas, with two of these areas, ‘medical, psychological and psychosocial improvements’ and ‘lethal means restriction’, being specifically applicable within health care. The prevention strategy recommends early interventions with a focus on treating depression, restricted prescriptions of sleeping pills, and increased use of new less-toxic antidepressants [2]. However, suicide still accounts for the deaths of around 1500 individuals per year in Sweden, with rates of 15.75 suicides per 100,000 males and 7.09 per 100,000 females having been reported in 2016 [3]. The suicide rate of ≥15 per 100,000 is among the highest rates reported worldwide [4]. Suicide rates and suicide attempts are often associated with a diagnosis of severe psychiatric or somatic illness [4, 5], and vary across lifetime and gender [4]. It is important to note that around 90% of individuals who commit suicide have a documented history of a psychiatric disease [6–8]. The psychiatric disorders with a high lifetime risk of suicide are the affective disorders [9], particularly bipolar disorder [10], alcohol or substance use disorders [11, 12], schizophrenia [13, 14], and personality disorders [15, 16]. Indeed, previous research has emphasized treatment interventions for psychiatric illness to reduce suicidal ideation and suicidal behavior. Psychopharmacological treatment [17], cognitive therapy for suicide prevention (CT-SP) [18], cognitive behavioral therapy (CBT) [1, 19], dialectical behavior therapy (DBT) [20], and electroconvulsive therapy (ECT) [21, 22] have all been found to be effective for preventing suicide. In addition, research also suggests that repeated suicide attempts can be significantly reduced or prevented by rather short psychotherapeutic interventions [23]. Despite promising results [19, 24], further research into suicide as an outcome is warranted to draw firm conclusions concerning the impact of different interventions on the suicide rate [1, 5, 25]. In particular, more studies are needed on treatments that combine psychotropic medication and psychotherapy to refine treatment recommendations for suicidal behavior [26]. Hence, the necessary requirements for successful interventions for patients with suicidal behavior or suicidal ideation are not yet fully understood [27].

Furthermore, lifetime suicide risk has been suggested to be hierarchical, meaning that psychiatric inpatients have the highest risk of suicide, while the risk is lower in psychiatric outpatients, and even lower in individuals with no history of contact with psychiatric services [9]. Suicide risk is also higher shortly after the onset of psychiatric illness and among recently hospitalized psychiatric patients with suicidal ideation or a history of suicide attempts [9, 28]. Additionally, patients admitted to psychiatric inpatient care are at higher risk of suicide shortly after admission, during hospitalization, during periods of authorized hospital leave, and at discharge, with the suicide risk remaining elevated up to 12 months post-discharge [29–31]. In relation to this, a study by Appleby et al. showed that a reduction in care efforts during the 12 months prior to suicide was observed significantly more often for suicide victims than for controls [32]. The suicide cases more often had reduced outpatient appointment frequencies, less supervision, and lower drug doses than the controls, with the authors finding strong associations between each of these three factors and completed suicide. These findings have also been confirmed in more recent research [33]. Hence, maintained and regular contact with psychiatric services and the avoidance of abrupt cessation of mental health care appear to lower the risk of suicide [5]. In addition, frequent follow-ups or outreach, especially after missed mental health visits, have been shown to reduce repeated suicide attempts [24].

In summary, research shows that suicidal behavior can be prevented through the application of appropriate medical, psychotherapeutic, or psychosocial interventions, and that reduced health care consumption is associated with suicide. However, important limitations of the previous research are that the above-mentioned factors were largely examined separately, rather than in combination, and the studies did not use control groups drawn from patients with the same diagnoses. To determine whether these different factors are independently associated with suicide, investigation of multiple associated factors is needed using data from suicide cases and adequately matched control subjects.

The overarching aim of the current study was to examine whether completed suicide in psychiatric patients in a Swedish population was associated with the quantity and nature of previous medical and psychosocial treatment interventions, with this being accomplished by comparing cases of suicide with matched control psychiatric patients.

The study tested the following two hypotheses:

1. *Controls have a higher frequency of psychiatric outpatient visits than suicide cases.*
2. *Controls have a higher occurrence of psychosocial treatment interventions than suicide cases.*

## Method

### Study design

This study used a retrospective psychiatry-based case-control design. Data from the period 1 January 2007 to 31 December 2013 were obtained from the Swedish National Cause of Death Registry [3], psychiatric and medical records, and the statistics of the Swedish total population [34].

### Study setting

The study catchment area (Örebro County) covers a population of 285,395, with a mix of urban and rural areas. In 2013, the region had suicide rates of 21.7 suicides per 100,000 male inhabitants and nine suicides per 100,000 female inhabitants. These rates were higher than the Swedish national rates for both males (16.2 per 100,000) and females (7.5 per 100,000) during the same period [35]. The specialist psychiatric unit belongs to a general university hospital with 918 beds, of which 136 are part of the psychiatric care unit. In 2013, the psychiatric units offered 40,020 days of inpatient psychiatric care and 131,137 outpatient department visits. Persons outside specialist psychiatry with visits only to community services outside of health care, or those at private clinics, are not included in this study.

### Sample and participant selection

#### Suicide cases

Suicide cases were identified using the Swedish National Cause of Death Registry. According to the registry, during the period 1 January 2007 to 31 December 2013, a total of 339 individuals (69.3% men) from Örebro County died secondary to suicide (codes X60–X84) or undetermined intent (codes Y10–Y34) classified in accordance with the International Statistical Classification of Diseases and Related Health Problems, 10th Revision (ICD-10) [35]. The national identification numbers of these individuals were used to search the electronic psychiatric medical records of the University Hospital of Örebro. This revealed that 154 (45.4%) of these 339 individuals had received psychiatric care at this regional centre during the 2 years prior to their death. These 154 individuals (65.6% men) were therefore included in the present study as suicide cases. Cases classified as uncertain suicides were also included, as their exclusion may have led to an underestimation of the suicide rate. The national statistics on causes of death include cases of uncertain cause of death as possible suicides, as analyses indicate that the majority of uncertain cases are probable suicides [3, 36]. In the subsequent text, the term suicide refers to both definite suicide and uncertain suicide.

##### Controls

The 154 control subjects were identified through the electronic psychiatric medical records of the University Hospital of Örebro during the matching procedure (see next section).

##### Case-control matching procedure

A hospital statistician outside the research team personnel matched the suicide cases with control subjects on the basis of the following: 1) a history of contact with psychiatric services in the year that the suicide case died, 2) age, 3) sex, and 4) primary psychiatric diagnosis. For the primary psychiatric diagnosis, consistency was required in terms of the first two or three digits of the respective ICD-10 code (e.g., F32.2 or F32). When applicable, efforts were made to control for a comorbid diagnosis of psychoactive substance use disorder (F10-F19), as comorbid substance use disorders increase the risk for suicide [37, 38]. For suicide cases below the age of 25 years, a control of similar age (± 2 years) was sought. In older suicide cases, controls with a maximum age difference of 2 years were primarily sought; however, a maximum age difference of 10 years was accepted.

A total of 113 (73.4%) suicide case-matched control pairs met the stringent primary diagnosis matching criteria (e.g., F32.2). Twenty-five (16.2%) pairs were matched on the basis of two digits (e.g., F32), and two (1.3%) pairs were matched on the basis of a diagnostic cluster (e.g., Mood disorders F30–F39). Thirteen suicide cases (8.5%) lacked a primary psychiatric diagnosis. Of these, 12 were matched with controls without psychiatric diagnoses, while a control with a primary diagnosis of depression was selected as the optimal match for the remaining case (0.6%). A total of 15 suicide cases had a comorbid diagnosis of psychoactive substance use (F10–F19). Of these, nine were matched to a control with a similar comorbidity.

For suicide cases younger than 25 years, 16 out of 18 pairs (88.9%) were matched according to the study criteria. For the remaining two pairs, the age-interval was extended to ±3 years and ± 7 years. The majority (87.5%) of the 136 case-control pairs in the age group 25 years or older were matched according to the study criteria (i.e., a maximum age-interval of ±2 years); however, the age-interval was extended to ±3 years in seven pairs, ± 4 years in three pairs, and ± 5 years in five pairs. In the remaining pair, a 96-year-old suicide case was matched to an 85-year-old control.

The mean follow-up time in days was calculated from the date of the first psychiatric care contact included in the study to the date of suicide (for suicide cases), or to the date of last contact included in the study (for controls). The mean follow-up time was 446 days (SD 256.4) for suicide cases and 479 days (SD 254.1) for control subjects, with no significant difference being found between suicide cases and controls according to an independent *t*-test analysis (*t* = 1.13, df 306, *p* = 0.259).

### Measures

#### The Swedish National Cause of death registry

The suicide cases in this study were identified from the National Cause of Death registry. This contains information on the deaths of all Swedish citizens, including deaths occurring outside Sweden. The registry does not include information concerning the deaths of people seeking asylum, undocumented migrants, or visitors to Sweden. Information concerning suicide is based on death certificates. These are completed by a physician following a clinical or forensic autopsy, with the cause of death being classified in accordance with the ICD-10 codes [35]. The annual rate of missing data on causes of death for Swedish citizens is less than 2% [3]. For the purposes of the present study, the following data were collected: national identification number, municipality, date of death, underlying cause of death, and medical evaluation of whether death by self-harm was intentional or of uncertain intent.

##### Data from medical records for suicide cases and controls

Information concerning psychiatric outpatient visits and psychiatric and somatic admissions in the 2 year period prior to each suicide was retrospectively collected from electronic medical records. The data concerned all care received from the 1st January 2005 until the 31st December 2013. For example, for a suicide case with a date of death of 14th March 2007, data were collected from 14th March 2005 to 14th March 2007 inclusive. Care consumption for controls was included from the same year as the death of the case, and the data were collected for 2 years back in time.

For each suicide case and control subject, data-files were created from electronic psychiatric medical records, and if applicable, from the relevant somatic medical records. From these files, data were retrieved concerning the number of psychiatric outpatient visits, the number of psychiatric admissions, the total number of days of hospitalization (from admission to discharge) for each psychiatric admission, the total number of days spent as a psychiatric inpatient, the occurrence of somatic hospitalization, and the ICD-10 diagnoses within the 2 year study period.

##### Psychiatric interventions

Information concerning the interventions implemented during outpatient visits and/or inpatient care in psychiatry (e.g., psychological treatment, prescriptions of psychotropic drugs, and ECT) was gathered from the electronic medical records.

##### Somatic comorbidity and serious suicide attempts

Data on somatic hospitalization at a specialist medical unit together with diagnoses according to ICD-10 codes (see Appendix for ICD-codes) were used as indicators of somatic comorbidity, and somatic comorbidity was treated as a possible covariate in the analyses, as it is assumed to increase suicide risk. Somatic comorbidity did not include hospitalization secondary to injuries or suicide attempts. A serious suicide attempt was defined as any intentional attempt to end life that led to hospitalization at a somatic specialist medical unit (e.g., due to intoxication). The ICD-10 codes for previous suicide attempts are provided in the Appendix.

### Statistical analysis

Descriptive statistics were used to describe the distribution of psychiatric diagnoses, sex, and age in the suicide cases and control subjects. The chi-square test and Fisher’s exact test were used to test for significant case-control differences in the occurrence (yes/no) of outpatient or inpatient psychiatric care, somatic hospitalization due to comorbidity, somatic hospitalization due to previous serious suicide attempts, and specific psychiatric interventions. The Mann Whitney U-test was used for case-control comparisons involving non-normally distributed variables (e.g., the frequency of health care provision).

Treatment interventions/covariates for which case-control differences (*p* < 0.1) were found (see Table 2) were evaluated as predictors or covariates using stepwise forward logistic regression models (i.e., a selection method using the likelihood ratio) to examine their association with suicide. This analysis was used to compute odds ratios (ORs) and confidence intervals (CIs) with suicide (yes = 1, no = 0) as a binary dependent outcome variable. Correlations between the included interventions and covariates were examined with Spearman correlation analyses. All statistical analyses were performed using SPSS for windows version 22 (IBM, New York).

A power calculation was performed on the basis of assumptions of 25% exposure to treatment interventions in controls, an OR of 2, and a confidence level of 95%. This showed that to obtain a power of 80% for the detection of significant differences, a sample size of 154 patients per group was required. If the exposure to treatment among controls was only 5%, an OR > 3.2 would be required to detect significant differences with this sample size.

## Results

The majority of cases died within 2 weeks from their last care contact (median times of 12 days in men and 11 days in women). The distributions of age, sex, and primary psychiatric diagnoses according to ICD-10 [35] among the suicide cases and controls are summarized in Table 1. The distribution of comorbidities was similar for both groups, with a total of 15 suicide cases and 14 controls having a comorbid substance use disorder (F10–F19). In terms of socio-demographics, the suicide cases and controls did not differ significantly in education, living situation, or occupation (see Table 1).

**Table 1:** Characteristics of the study population after matching for age, sex, and psychiatric diagnosis, and results of chi-square-tests/*t*-tests (suicide cases versus controls)

	Suicide cases *n* = 101 Men^4^	Controls n = 101 Men^4^	Suicide cases *n* = 53 Women^4^	Controls n = 53 Women^4^	All suicide cases *n* = 154	All controls n = 154	Test statistic, p
Mean age: years (SD^5^) Range	45.9 (17.6) 18–84	45.9 (17.4) 17–84	49.6 (16.4) 13–96	49.4 (15.5) 13–85	47.1 (17.2) 13–96	47.1 (16.8) 13–85	*t*: −0.040, *p* = .97^6^
Educational level^*1,8*^ n (%)							χ^2^: 4.085, df 2, *p* = .13^7^
Low	28 (27.7)	44 (43.6)	15 (28.3)	16 (30.8)	43 (27.9)	60 (39.0)	
Medium	54 (53.5)	42 (41.6)	25 (47.2)	25 (48.1)	79 (51.3)	67 (43.5)	
High	18 (17.8)	14 (13.9)	11 (0.8)	11 (21.2)	29 (18.8)	25 (16.2)	
Occupational status^8^ n (%)							χ^2^: 0.14, df 1, *p* = .71^7^
Employed	37 (36.6)	29 (28.7)	12 (22.6)	17 (32.1)	49 (32.0)	46 (30.1)	
Unemployed	64 (63.4)	72 (71.3)	40 (75.5)	35 (66.0)	104 (67.5)	107 (69.5)	
Family status^8^ n (%)							χ^2^: 3.23, df 3, *p* = .36^7^
Cohabiting with partner (no children)	12 (11.9)	12 (11.9)	8 (15.1)	11 (20.8)	20 (13.0)	23 (14.9)	
Cohabiting with partner and children	14 (13.9)	20 (19.8)	4 (7.5)	8 (15.1)	18 (11.7)	28 (18.2)	
Single parent with children	11 (10.9)	5 (5.0)	5 (9.4)	8 (15.1)	16 (10.4)	13 (8.4)	
Living alone	64 (63.4)	64 (63.4)	35 (66.0)	25 (47.2)	99 (64.3)	89 (57.8)	
Diagnoses used for matching^2^ n (%) Depressive disorders (F32–F33, F34.1)	27 (26.7)	28 (27.7)	24^2^ (45.3)	24 (45.3)	51 (33.1)	52 (33.8)	χ^2^: 0.047, df 7, *p* = 1.00^7^
Mental and behavioural disorders due to psychoactive substance use (F10–F19)	28 (27.7)	28 (27.7)	5 (9.4)	5 (9.4)	33 (21.4)	33 (21.4)	
Neurotic, stress-related, and somatoform disorders (F40–F48)	13 (12.9)	13 (12.9)	8 (15.1)	8 (15.1)	21 (13.6)	21 (13.6)	
Schizophrenia, schizotypal, and delusional disorder (F20–29)	9 (8.9)	9 (8.9)	5 (9.4)	5 (9.4)	14 (9.1)	14 (9.1)	
Disorder of adult personality and behaviour (F60–F69)	7 (6.9)	7 (6.9)	4 (7.5)	4^2^ (7.5)	11 (7.1)	11 (7.1)	
Bipolar disorder (F31)	3^2^ (3)	3 (3)	4 (7.5)	4 (7.5)	7 (4.5)	7 (4.5)	
Disorder of psychological development / behavioral and emotional disorders (F80–89, F90–98)	3 (3)	3 (3)	0	0	3 (1.9)	3 (1.9)	
Psychiatric examination^3^	11 (10.9)	10 (9.9)	3 (5.7)	3 (5.7)	14 (9.1)	13 (8.4)	

^1^ Low: elementary school or grade 1–9; Medium: high school or other education below university level; High: university or university college

^2^ Diagnoses were mental and behavioural disorders referring to chapter V in the International Statistical Classification of Diseases, 10th Revision classifications (ICD-10)

The diagnoses were registered as primary diagnoses in all but two cases and one control, where the secondary diagnoses were used (see ^2^ in the suicide case and control columns above)

^3^ Persons who received psychiatric care without being assigned a primary psychiatric diagnosis

^4^ A comorbid diagnosis of psychoactive substance use (F10-F19) was present in 22 patients (five male and seven female suicide cases; five male and five female controls)

^5^ SD = standard deviation

^6^ Results from Independent Samples *t*-test

^7^ Results from Pearson Chi-Square Tests

^8^ Number of patients with missing data: educational level: 5, occupational status: 2, family status: 2

### Provision of psychiatric care

Medical and psychological assessments, supportive conversation, and efforts to improve everyday functioning were regular contents of both psychiatric inpatient and outpatient care. During the 2 years before the suicide, outpatient psychiatric care was received by 305 patients (305/308, 99%) in the total cohort, and inpatient care was received by 155 patients (155/308, 50.3%) in psychiatric specialist units. Three of the suicide cases received inpatient care only, while all controls had at least one outpatient visit. Similar proportions of the occurrence (yes/no) of outpatient care were observed in the suicide case and control groups; however, during the 2 years before death, suicide cases made a significantly lower number of visits to outpatient psychiatry than controls. On average, the control subjects attended five times as many outpatient visits as suicide cases. No significant case-control differences were found in the occurrence of inpatient psychiatric care, involuntary inpatient admissions, the number of psychiatric inpatient admissions, or the total days of hospitalization (for details see Table 2).

**Table 2:** Case-control comparisons of clinical variables and somatic inpatient care because of somatic comorbidity or serious suicide attempts

	Suicide cases n = 154	Controls *n* = 154	
	n (%)^11^	n (%)^11^	Test statistic, p
Provision of psychiatric care			
Outpatient visits (yes)	151 (98.1)	154 (100)	F, *p* = .248^9^
Inpatient admissions (yes)	80 (51.9)	75 (48.7)	χ^2^: 0.32, df 1, *p* = .569^10^
of which involuntary admissions (yes)	15 (9.7)	9 (5.8)	χ^2^: 1.63, df 1, *p* = .202^10^
Number of outpatient visits, Md^1^	4 (1–217)	19.5 (1–209)	U, *p* = .001^9^
Number of admissions, Md^1^	2 (1–11)	2 (1–22)	U, *p* = .954^11^
Hospitalization days, Md^1^	20.5 (1–693)	16 (1–191)	U, *p* = .443^11^
Psychiatric interventions			
Intervention forms			
Combination treatment (yes) ^2^	17 (11.0)	41 (26.6)	χ^2^: 12.27, df 3, *p* = .007^10^
Psychotherapy (yes)^3^	2 (0.9)	2 (0.9)	
Psychotropics (yes)^4^	119 (77.2)	98 (63.6)	
Other (yes)^5^	16 (10.4)	13 (8.4)	
Number of psychotherapy sessions, Md^1^	5 (1–47)	13 (1–142)	U, *p* = .504^11^
CBT (including DBT)	8 (5.2)	17 (11)	χ^2^: 3.53, df 1, *p* = .060^10^
PDT	12 (7.8)	14 (9.1)	χ^2^: 0.17, df 1, *p* = .838^10^
Other structured psychotherapy	6 (3.9)	18 (11.7)	χ^2^: 6.51, df 1, *p* = .011^10^
Electroconvulsive therapy^6^	13 (8.4)	13 (8.4)	χ^2^: 0.00, df 1, *p* = 1.00^10^
Provision of somatic inpatient care			
Somatic comorbidity (yes)^7^	50 (32.5)	30 (19.5)	χ^2^: 6.75, df 1, *p* = .009^10^
Serious suicide attempts^8^	30 (19.5)	14 (9.1)	χ^2^: 6.79, df 1, p = .009^10^

^1^ Md: Median (minimum to maximum)

^2^ Combination treatment: psychotropics and psychotherapy (CBT, DBT, PDT or other structured psychotherapy)

^3^ Mono treatment: psychotherapy without psychotropics

^4^ Mono treatment: psychotropics without psychotherapy

^5^ Other supportive interventions

^6^ All patients with ECT were prescribed psychotropic drugs. Two ECT cases (controls) also received psychotherapy

^7^ Somatic specialist medical inpatient treatment because of somatic comorbidity. Diagnostic ICD-10 codes provided in the Appendix

^8^ Somatic specialist medical inpatient treatment because of serious suicide attempts. Diagnostic ICD-10 codes provided in the Appendix

^9^ Results from Fisher’s Exact Test (F)

^9^ Results from Pearson Chi-Square Tests (χ^2^)

^10^ Results from Mann Whitney U-test (U)

^11^ n (%) = number (percent) of patients in each group

### Psychiatric interventions

Psychiatric interventions included prescriptions of psychotropic drugs, ECT, and psychotherapeutic interventions such as CBT, DBT, psychodynamic therapy (PDT), or other structured psychological treatment approaches (e.g., integrated psychotherapy using a mix of several psychotherapeutic approaches to meet the needs of the patient). These different interventions were received by 279 (90.6%) patients in the total sample (suicide cases: 89.6%, controls: 91.6%). Case-control comparisons revealed no differences in the occurrence of ECT or psychotherapy without combined use of psychotropic drugs. By contrast, significant case-control differences were found in the provision of combination therapy (i.e., both psychotherapy and prescription of psychotropic drugs), which was significantly more frequent in controls, while mono-therapy with psychotropic drugs without psychotherapy was significantly more frequent in the suicide cases. Between group comparisons in the type of psychotherapy received showed ‘other structured psychotherapy’ to be significantly more frequent in controls, while the number of psychotherapy sessions received did not differ significantly between the groups (for details see Table 2).

### Provision of somatic inpatient care

A significant case-control difference was found for somatic hospitalization. Around one-third of suicide cases and a fifth of controls had a somatic comorbidity that required specialist inpatient medical treatment (Table 2). In the suicide cases, the most common somatic comorbid diagnoses were diseases of the musculoskeletal system (*n* = 10, 6.5%, ICD-10: chapter M) and diseases of the circulatory system (*n* = 8, 5.2%, ICD-10: chapter I). In the controls, the most common somatic comorbid diagnoses were diseases of the musculoskeletal system (*n* = 5, 3.2%, ICD-10: chapter M) and diseases of the digestive system (n = 5, 3.2%, ICD-10: chapter K). The somatic diagnoses in suicide cases and controls are specified in the Appendix. Serious suicide attempts that required somatic inpatient treatment were significantly more prevalent among the suicide cases than the controls during the 2 years before death. The suicide attempt diagnoses are specified in the Appendix. The distribution between groups is shown in Table 2.

### Factors and treatment interventions associated with suicide

The psychiatric interventions and covariates showing associations (*p* < 0.1) with suicide (see Table 2) in the present cohort were analyzed using a stepwise forward selection method (likelihood ratio) in a logistic regression analysis. The full model included the following six variables: ‘number of visits in outpatient psychiatry’, ‘intervention form’ (i.e., psychotropic prescription without psychotherapy, combination therapy, psychotherapy without psychotropics, other supportive interventions), ‘CBT’ (including DBT), ‘other structured psychotherapy’, ‘somatic hospitalization due to serious suicide attempt’, and ‘somatic hospitalization due to somatic comorbidity’. The results from the full stepwise logistic regression model (Table 3) indicated that a high frequency of outpatient visits was significantly negatively associated with suicide, while other structured psychotherapy approaches did not reach significance (*p* = .056). The occurrence of serious suicide attempts was significantly associated with successful suicide. This full model explained 9.9% of the variance (Table 3).

**Table 3:** Results from a binominal logistic regression (full model) with a stepwise forward selection method (likelihood ratio). Suicide cases, *n* = 154; Controls, n = 154

Full model*	B (SE)	Wald	Sig.	OR (CI)	95% CI for OR	
					Lower	Upper
Number of outpatient visits	−0.012 (0.004)	7.908	.005	0.988	0.980	0.996
Serious suicide attempt (yes)^1^	1.043 (0.364)	8.233	.004	2.839	1.392	5.790
Other structured psychotherapy	−0.970 (0.509)	3.641	.056	0.379	0.140	1.027

Note: The dependent outcome variable *‘Suicide’* was dichotomized (Yes = 1, No = 0)

Abbreviations: B: beta coefficient, SE: standard error, OR: odds ratio / expected beta coefficient, CI: confidence interval for OR. For each step, the entry testing was based on the significance of the score statistic, and removal testing was based on the probability of a likelihood-ratio statistic. Degrees of freedom (df): 1 in all steps, except for ‘Intervention form’ (df = 3)

^1^ Somatic specialist medical inpatient treatment due to serious suicide attempt; for diagnostic ICD-10 codes, see Appendix

^*^Full model: The six independent predictor variables/covariates entered in the stepwise forward analysis were: ‘Number of visits’ (continuous variable), ‘Intervention form’ (categorical variable: Combination therapy, Psychotherapy, Psychotropics (*reference*), Other), ‘CBT’ (incl. DBT), and ‘Other structured psychotherapy’, ‘Serious suicide attempt’, and ‘Somatic comorbidity’ (dummy coded [Yes = 1, No = 0] variables

Full model fit: R^2^ = 0.099 (Nagelkerke). Model χ2 = 23.818; *p* = .001

Spearman correlation analyses revealed significant (*p* < .01) correlations between the ‘number of outpatient visits’ and the various psychiatric interventions (*r* = .27 to .46). To further investigate the impact of other variables on suicide, we excluded the strongest variable ‘number of outpatient visits’ from an additional stepwise logistic regression model, but included all the other variables used in the full model. The results from this model indicated that the intervention consisting of combined pharmacotherapy and psychotherapy treatment was the only intervention that was significantly negatively associated with suicide, while previous severe suicide attempts remained significantly associated with suicide. This additional model explained 8.4% of the variance (Table 4).

**Table 4:** Results from a binominal logistic regression (additional model) with a stepwise forward selection method (likelihood ratio). Suicide cases, n = 154; Controls, n = 154

Additional model**	B (SE)	Wald	Sig.	OR (CI)	95% CI for OR	
					Lower	Upper
Intervention form:	–	12.153	.007	–	–	–
Combination treatment ^2^	−1.113 (0.325)	11.746	.001	0.329	0.174	0.621
Psychotherapy^3^	−0.065 (1.010)	0.004	.949	0.937	0.129	6.788
Psychotropics^4^				*reference*		
Other^5^	0.053 (0.401)	0.017	.895	1.054	0.480	2.316
Serious suicide attempt (yes)^1^	0.950 (0.357)	7.074	.008	2.585	1.284	5.206

Note: The dependent outcome variable *‘Suicide’* was dichotomized (Yes = 1, No = 0)

Abbreviations: B: beta coefficient, SE: standard error, OR: odds ratio / expected beta coefficient, CI: confidence interval for OR. For each step, the entry testing was based on the significance of the score statistic, and removal testing was based on the probability of a likelihood-ratio statistic. Degrees of freedom (df): 1 in all steps, except for ‘Intervention form’ (df = 3)

^1^ Somatic specialist medical inpatient treatment due to serious suicide attempt; for diagnostic ICD-10 codes, see Appendix

^2^ Combination treatment: psychotropics and psychotherapy (CBT, DBT, PDT, or other structured psychotherapy)

^3^ Mono treatment: psychotherapy without psychotropics

^4^ Mono treatment: psychotropics without psychotherapy

^5^ Other interventions (e.g., supportive conversation)

**Additional model: Five predictor variables/covariates entered in the stepwise forward analysis: ‘Intervention form’, ‘CBT’ (incl. DBT), ‘Other structured psychotherapy’, ‘Serious suicide attempt’, and ‘Somatic comorbidity’. The variable ‘Number of outpatient visits’ was excluded from this model. Additional model fit: R^2^ = 0.084 (Nagelkerke). Model χ2 = 20.155; p = .001

## Discussion

The results of the present case-control comparisons indicate that a higher frequency of outpatient visits and provision of combination therapy were significantly negatively associated with suicide, and a history of serious suicide attempts and somatic comorbidity were significantly more frequent in the suicide cases. The results will be further discussed below.

Over the 7-year inclusion period, about 45% of total suicide cases in the county had treatment contact with specialist psychiatric care before suicide; this is the same amount as reported by Ahmedani et al. [33], but somewhat higher than the proportion of around a third reported by Luoma et al. [39]. The suicide cases received fewer psychiatric treatment interventions and attended fewer outpatient visits than the control group. No case-control differences were found in the number of psychiatric inpatient admissions or the total number of days of psychiatric inpatient care. Compared with suicide cases, a higher proportion of controls received intervention in the form of systematic psychological treatment in combination with psychotropic medication. One possible explanation for this finding is that the suicide cases died during the early course of their disease, i.e., before they had commenced or completed treatment. However, this finding could also indicate that, for various reasons, the suicidal patients received fewer interventions demonstrated to be effective in reducing suicidal behavior. These data support our first hypothesis that controls have a higher frequency of psychiatric outpatient visits than suicide cases. The results also partly support our second hypothesis that controls have a higher occurrence of medical and psychosocial treatment interventions than suicide cases, with the exception of medical treatment as mono-therapy, which was significantly more frequent in the suicide cases, and the occurrence of ECT, which was equally prevalent in both groups. The number of psychotherapy sessions did not differ between the groups, and hence did not seem to protect from suicide as a single factor. However, as part of the combination treatment (psychotherapy and medication), the presence (rather than quantity) of psychotherapy seemed to lower the risk of suicide. In addition, we found somatic comorbidity that required somatic hospitalization to be significantly more prevalent among suicide cases. This is in line with other studies [8, 29, 40–43] reporting an elevated risk of suicidal behavior or completed suicide with the occurrence of a somatic disease. To reduce the risk of suicide in patients with somatic comorbidity, it is important that psychiatric interventions (for example, appropriate pain management) are offered close in time to critical events in the course of the somatic illness, i.e., at initial diagnosis, times of deterioration or relapse, and the transition to palliative care [42, 44, 45]. Somatic comorbidity, used as a covariate in our study, did not independently contribute to increasing suicide, nor was it identified as a unique factor associated with suicide in the multivariate analysis. By contrast, a severe suicide attempt during the last 2 years before suicide was independently associated with suicide, which is in accord with several other studies [5, 9, 28, 42, 46].

The suicides in this study occurred in a median time of 11 (females) or 12 days (males) from the patients’ last time of contact with psychiatric outpatient services. This might indicate that suicidal patients require frequent outpatient visits or close follow-up. This points in the same direction as earlier research [5] and the global suicide prevention strategy of the World Health Organization (WHO) [47], which emphasizes follow-up as one of the most important methods of suicide prevention. Continuation of treatment beyond the stage of clinical recovery has also been found to be protective in patients with a high risk of suicide [32].

### Strength and limitations

The present study has several strengths. First, suicide data were extracted from the National Causes of Death Registry, thus ensuring that all suicide cases within Örebro County were included. Second, suicide cases and controls were matched according to factors previously associated with suicide risk, i.e., age, sex, and primary psychiatric diagnosis, and both suicide cases and controls were current or past patients within psychiatry [5, 48]. The successful matching using stringent diagnostic criteria (three digits) on primary ICD-10 F-diagnoses for the majority (73.4%) of the sample is a strength. This means that in the majority of the suicide cases, the levels of severity were also taken into account through matching with a control with a similar severity of symptoms (for example, the third diagnosis digit specifies if a depression**/**recurring depression was mild, moderate, or severe). Other facts that indicate that important aspects of the suicide case and control groups were similar are that the groups did not differ significantly in the distribution of comorbid substance use, educational level, living situation, employment, or follow-up time. A third strength was that the retrospective design made it possible to collect data for all patients who died from suicide, regardless of the level of suicide risk before death. In randomized controlled trials, patients with an immediate suicide risk are often excluded for patient safety reasons [49]. A fourth strength was the use of data from electronic psychiatric medical records. The WHO [47] has emphasized the need to use data from hospital-based systems to develop strategies to prevent both suicide attempts and completed suicides. The electronic psychiatric medical records contain important data on treatment interventions implemented not only by psychiatrists, but also by psychologists, psychotherapists, psychiatric nurses, and other medical personnel. The use of such data (so far not collected in the Swedish national patient register) enabled us to examine whether the patients benefited from a combination treatment. Fifth, two major risk factors for suicide were controlled for as covariates in the forward logistic regression analysis.

The present study also has limitations. First, the associations between suicide and fewer visits to outpatient psychiatry or less combination treatment might be explained by factors not possible to control or match for. For example, patients who are suicidal may be offered more frequent visits, but may choose to decline further visits or interventions for unknown reasons. A poor adherence to treatment is identified as another factor that increases the risk of suicide [13, 32]. Alternatively, fewer visits or less combination treatments could also be a result of a clinical decision where a psychologist may be reluctant to offer psychotherapy to severely symptomatic and functionally impaired patients because of their potentially high suicide risk. Psychotherapy is one of the drivers of frequent outpatient visits in psychiatry. Severely functionally impaired patients may be offered support by community services outside of psychiatric care, and such data are not included in the psychiatric records. We found no way to retrospectively collect data on the level of function in suicide cases, and were therefore unable to compare the groups in this respect. Moreover, help-seeking behavior among patients might influence both the frequency of psychiatric outpatient visits and the risk of suicide. Therefore, selection effects may have influenced the negative associations found between suicide and more frequent outpatient visits and interventions. In addition, the occurrence or number of psychiatric inpatient admissions, involuntary treatment periods, or differences in follow-up time between suicide cases and controls may have affected the possible number of outpatient visits. However, the present study found no significant differences between the groups in this regard, and it does not therefore explain the limited numbers of outpatient visits among the suicide cases. Another limitation of our study is that it was not possible to match for all comorbidities or other factors that are assumed to increase the suicide risk; for example, levels of comorbid anxiety. A recent large prospective study of comorbid anxiety disorders in mood disorder patients found no significant differences in survival curves for patients with or without anxiety comorbidity [50]. However, other well-known risk factors for suicide, such as previous suicide attempts and somatic comorbidity, which differed significantly between our suicide cases and controls, were controlled for in the multivariate analysis. Furthermore, we lacked information on treatments and outpatient visits in clinics outside of the Region Örebro County, including private psychotherapy. However, all outpatient visits within the county health care system are likely to have been recorded in the electronic medical records, although some data regarding the content of some visits may have been missing. We also lack information about referrals. Nevertheless, the quality of the medical record content is unlikely to have differed between cases and controls. Another limitation was the relatively small number of patients included in the study, which precludes firm conclusions regarding the effects of certain interventions, including ECT. Participants were not matched for time passed since first contact with psychiatry, which may limit the comparability between the groups. Finally, suicide risk may have been influenced by non-health care system factors, such as negative life stressors shortly before death [51–54]. Although such factors were beyond the scope of the present analyses, they could add valuable knowledge if they were to be included in future research.

## Conclusions

In the studied sample, completed suicide was significantly negatively associated with the quantity of psychiatric outpatient visits and the presence of psychotropic medication combined with psychotherapy, but not with the number of inpatient treatments or the quantity of psychotherapy sessions. Underlying causes for these differences need to be further studied before any firm conclusion can be drawn on whether the studied interventions may have a protective effect against suicide, or whether the associations may have been attributable to other factors that were not possible to control for in this study.

## Data Availability

The dataset analyzed during the current study is available from the corresponding author on request.

## Appendix

### ICD-10 codes used in the study

*ICD-10 codes in suicide attempts that required somatic inpatient care (cases).*

**T060.**

**T148.**

**T213, T292.**

**T312.**

**T424, T427, T432, T438, T439.**

**T509, T519, T559,**

**T659.**

**T719.**

***ICD-10 codes in suicide attempts that required somatic inpatient care (controls).***

**T424, T425, T427, T432, T433, T436,**

**T509, T510, T519.**

**Somatic comorbidity, ICD-10 codes during somatic hospitalization (cases).**

A: A414.

B: B182.

C: C549, C569, C649.

E: E871.

G: G122 (2 patients), G250, G442, G931.

H: H811.

I: I109, I210, I269, I479, I489, I495.

J: J441, J690.

K: K250, K400, K550, K859, K860.

L: L890.

M: M059, M161, M170, M245, M329, M511, M544, M545, M549, M628.

N: N409.

O: O049.

R: R074, R119, R509, R559, R568, R568.

**Somatic comorbidity, ICD-10 codes during somatic inpatient hospitalization (controls).**

A: A045.

B: B182.

C: C649.

D: D259.

G: G403, G431, G459.

H: H811

I: I200, I269, I639J: J159, J441, J939.

K: K359, K501, K567, K579, K829.

M: M059, M171, M191, M243, M462.

R: R074, R104, R401, R568.

## Abbreviations

CBT: Cognitive behavioral therapy
CT-SP: Cognitive therapy for suicide prevention
DBT: Dialectical behavior therapy
ECT: Electroconvulsive therapy
ICD-10: International Statistical Classification of Diseases and Related Health Problems,10th Revision
PDT: Psychodynamic therapy
